# Prognostic value of LAT-1 status in solid cancer: A systematic review and meta-analysis

**DOI:** 10.1371/journal.pone.0233629

**Published:** 2020-05-29

**Authors:** Jing-jing Lu, Ping Li, Yong Yang, Le Wang, Yan Zhang, Jia-yao Zhu, Xiao-ren Zhu, Min-bin Chen

**Affiliations:** 1 Department of Oncology, Affiliated Kunshan Hospital of Jiangsu University, Kunshan, Jiangsu, China; 2 Department of Gastrointestinal Surgery, Affiliated Kunshan Hospital of Jiangsu University, Kunshan, Jiangsu, China; Chang Gung Memorial Hospital at Linkou, TAIWAN

## Abstract

**Background:**

The expression of the L-type amino acid transporter 1 (LAT1) plays a significant role in tumor progression. However, it remains unclear whether high LAT1 expression correlates with poor prognosis of solid tumor patients. Here, we conducted a meta-analysis to assess the potential of LAT1 in predicting the prognosis of tumor patients.

**Methods and findings:**

A total of 4,579 cases were analyzed from 35 qualified studies. In patients with solid tumors, elevated expression of LAT1 is associated with poor prognosis (overall survival [OS]: pooled hazard ratio (HR) = 1.848, 95% confidence interval (CI) = 1.620–2.108, *P <* 0.001; disease free survival [DFS]: pooled HR = 1.923, 95% CI = 1.585–2.333, *P <* 0.001; progression free survival [PFS]: pooled HR = 1.345, 95% CI = 1.133–1.597, *P* = 0.001). Furthermore, in subgroup analysis, we found an association between high LAT1 expression and poor OS in non-small cell lung cancer (HR = 1.554, 95% CI = 1.345–1.794, *P* < 0.001), pancreatic cancer (HR = 2.052, 95% CI = 1.613–2.724, *P* < 0.001) and biliary tract cancer (HR = 2.253, 95% CI = 1.562–3.227, P < 0.001).

**Conclusion:**

The results of this meta-analysis indicate the reliability and potential of using LAT1 expression as a predictive biomarker in solid cancers prior to treatment. However, further studies with larger sample sizes would be beneficial for fully evaluating the predictive value of LAT1 expression for clinical applications.

## Introduction

The L-type amino acid transporter 1 (LAT1) is a membrane protein responsible for transporting neutral amino acids, including phenylalanine leucine, valine, etc., as a part of system L for cellular intake of nutrients [1]. LAT1, also known as 4F2 light chain, is connected through a disulfide bond to the membrane-spanning 4F2 heavy chain (CD98) for its functional expression as part of a heterodimeric complex in the plasma membrane [2, 3]. It has been previously reported that LAT1 is associated with the prognosis of tumors.

Amino acid transporters are essential for cell growth and proliferation [1, 4]. The upregulation of LAT1 facilitates the growth of tumor cells [4–9], while selective inhibition of LAT1 inhibits the growth and proliferation of tumor cells [10–16]. It has been reported that LAT1 expression levels positively correlate with cell proliferation (Ki-67 labeling index), p53 expression, VEGF levels as well as poor prognosis of patients in a variety of solid cancers [8, 9, 17–20]. LAT1 transports essential amino acids to provide nutrients for cancer cell growth. Moreover, LAT1 induces rapid dephosphorylation of mTORC1 effectors S6K1 and 4EBP1 through regulation of amino acids such as leucine to activate mTOR signaling pathway, promoting the occurrence of tumors [21, 22].

However, it remains unclear whether LAT1 expression is associated with a worse outcome across solid cancer patients. These conflicting results may be due to the small sample size among individual studies and limitations of current technology. Therefore, we conducted this meta-analysis to evaluate the prognostic value of LAT1 expression as a predictive biomarker with the goal of guiding clinicians and patients in determining the best course of treatment.

## Materials and methods

### Publication search

Primary literature was collected from electronic databases PubMed, EMBASE and Web of Science for the LAT1 studies until August 10, 2019, with the search terms: (LAT1 OR “L-type amino acid transporter 1” OR SLC7A5 OR 4F2lc OR CD98lc) AND (prognosis OR survival OR predict OR outcome) AND (neoplasms OR neoplasms OR cancer OR tumor OR carcinoma). All publications that met requirements were included as well as eligible studies identified within their references. References cited in the original studies were manually evaluated for inclusion/exclusion criteria. In the event that the study had ongoing investigation of the same patient pool, the largest sample size at the time was included for this meta-analysis.

### Inclusion and exclusion criteria

The studies to be involved in this research were determined using the subsequent criteria: (a) evaluated the prognostic status of LAT1 in cancer, (b) provided HRs with 95% confidence intervals (CIs) or provided calculation or charts that displayed clear statistical analysis, (c) divided LAT1 status into ‘positive’ and ‘negative’ or ‘high’ and ‘low’, (d) articles issued in English.

Exclusion criteria: (a) reviews, summary of conference, editorials, letters, case reports, and abstracts, (b) the experiments were conducted in vivo or in vitro, but research design was not patient-based, (c) studies without HRs, 95% CI, or outcomes defined by OS, DFS, PFS, or the K-M survival curves. (d) the follow-up duration was less than 36 months.

### Data extraction

Based on the above criteria, authors Lu and Li comprehensively collected relevant data from all qualified publications in a standardized manner. If a consensus could not be reached, the third author (Chen) independently assessed the data from the initial article to review the discrepancy and agreement was achieved through discussion. The study was based on three outcome endpoints: OS, DFS and PFS. We extracted the following information from the selected papers according to the criteria above, including the name of the first author, country of origin, year of publication, number of patients analyzed, antibody, cancer types, LAT1 detection method, cut-off value, endpoints and Newcastle-Ottawa Scale (NOS). The summary of the included studies can be found in Table 1. For articles plotting prognosis only with Kaplan-Meier curve, survival data was extracted with Engauge Digitiser V4.1, and then HRs and 95% CIs were estimated by Tierney's method [23]. NOS was used to assess the quality of all studies [24]. Studies with NOS scores ranging from 6 to 9 were used for quality control of methodology.

**Table 1:** Characteristics of studies included in the meta-analysis.

Author	Year	Country	Cases	Cancer type	Antibody	Method	Cut-off value	Endpoints	NOS
Imai, H. [33]	2009	Japan	282	non-small cell lung cancer	rabbit anti-LAT1 antibody (1:3200 dilution)	ICH	>10% of tumor cells	OS	7
Kaira, K. [20]	2008	Japan	321	non-small cell lung cancer	rabbit anti-LAT1 antibody (1:3200 dilution)	ICH	>10% of tumor cells	OS	8
Kaira, K. [34]	2009	Japan	139	pulmonary adenocarcinoma	rabbit anti-LAT1 antibody (1:3200 dilution)	ICH	>10% of tumor cells	OS	8
Kaira, K. [35]	2009	Japan	220	non-small cell lung cancer	rabbit anti-LAT1 antibody (1:3200 dilution)	ICH	>10% of tumor cells	OS	8
Kaira, K. [36]	2010	Japan	188	non-small cell lung cancer	rabbit anti-LAT1 antibody (1:3200 dilution)	ICH	>10% of tumor cells	OS	7
Kaira, K. [37]	2010	Japan	59	non-small cell lung cancer	rabbit anti-LAT1 antibody (1:3200 dilution)	ICH	>10% of tumor cells	OS	8
Furuya, M. [8]	2012	Japan	50	breast cancer	mouse anti-LAT1 antibody (1:1 dilution)	ICH	scores of 2, 3, 4	OS,DFS	7
Kaira, K. [42]	2012	Japan	97	pancreatic cancer	mouse anti-LAT1 antibody (1:3200 dilution)	ICH	scores of 3, 4	OS,PFS	8
Yanagisawa, N. [43]	2012	Japan	66	pancreatic ductal adenocarcinomas	anti-LAT1	ICH	scores of 6–9	OS	9
Kaira, K. [45]	2013	Japan	139	biliary tract cancer	mouse anti-LAT1 antibody (1:3200 dilution)	ICH	scores of 3, 4	OS,PFS	7
Kaira, K. [17]	2013	Japan	30	adenoid cystic carcinoma	mouse anti-LAT1 antibody (1:3200 dilution)	ICH	scores of 3, 4	OS,PFS	7
Isoda, A. [57]	2014	Japan	100	multiple myeloma	mouse anti-LAT1 antibody (1:3200 dilution)	ICH	scores of 2+, 3+	OS,PFS	8
Namikawa, M [48]	2015	Japan	84	hepatocellular carcinoma	mouse anti-LAT1 antibody (1:3200 dilution)	ICH	scores > 1	OS	7
Toyoda, M.[58]	2014	Japan	85	tongue cancer	mouse anti-LAT1 antibody (1:3200 dilution)	ICH	scores of 3, 4	OS,PFS	6
Yanagisawa, N.[46]	2014	Japan	134	bile duct adenocarcinomas	anti-LAT1	ICH	score of 9	OS	7
Kaira, K. [50]	2015	Japan	142	ovarian tumors	mouse anti-LAT1 antibody (1:800 dilution)	ICH	scores of 2, 3, 4	OS	8
Nikkuni, O. [59]	2015	Japan	73	laryngeal squamous cell carcinoma	mouse anti-LAT1 antibody (1:800 dilution)	ICH	scores of 4, 5	OS	7
Shimizu, A.[60]	2015	Japan	128	cutaneous melanoma	mouse anti-LAT1 antibody (1:800 dilution)	ICH	scores of 2, 3, 4	OS,DFS	8
Shimizu, A.[61]	2017	Japan	52	cutaneous angiosarcoma	mouse anti-LAT1 antibody (1:800 dilution)	ICH	scores of 3, 4	OS,PFS	8
Imai, H. [16]	2010	Japan	51	non-small cell lung cancer	rabbit anti-LAT1 antibody (1:3200 dilution)	ICH	>10% of tumor cells	OS	8
Ichinoe, M.[52]	2011	Japan	87	gastric carcinomas	anti-LAT1	ICH	scores of 6–9	OS	8
Kaira, K. [38]	2009	Japan	84	squamous cell carcinoma of the lung	rabbit anti-LAT1 antibody (1:3200 dilution)	ICH	>10% of tumor cells	OS	9
Kaira, K. [62]	2011	Japan	21	Malignant pleural mesothelioma	rabbit anti-LAT1 antibody (1:3200 dilution)	ICH	>10% of tumor cells	OS	7
Takeuchi, K. [39]	2010	Japan	295	non-small cell lung cancer	mouse anti-LAT1 antibody (1:20 dilution)	ICH	>10% of tumor cells	OS	8
Li, J. [49]	2013	China	148	hepatocellular	goat anti-LAT1	ICH	Si > 4	OS	8
Yazawa, T.[40]	2015	Japan	222	lung adenocarcinoma	mouse anti-LAT1 antibody (1:3200 dilution)	ICH	scores ≥ 2	OS	9
Yanagisawa, N. [54]	2015	Japan	109	prostatic cancer	mouse anti-LAT1	ICH	scores of 4–9	OS	8
Ichinoe, M. [53]	2015	Japan	64	gastric carcinoma	anti-LAT1	ICH	scores of 6–9	OS	8
Yothaisong, S. [47]	2015	Thailand	178	cholangiocarcinogenesis	anti-LAT1	ICH	scores ≥ 4	OS	8
Altan,B. [44]	2018	Japan	110	pancreatic ductal adenocarcinoma	rabbit anti-LAT1 antibody (1:5000 dilution)	ICH	scores of 3, 4	OS,DFS	8
Hayase, S. [55]	2017	Japan	210	colorectal cancer	mouse anti-LAT1 antibody (1:200 dilution)	ICH	scores of 3, 4	OS	9
Kaira, K. [41]	2019	Japan	105	pulmonary pleomorphic carcinoma	rabbit anti-LAT1 antibody (1:5000 dilution)	ICH	scores of 4	OS,DFS	8
Ogawa, H. [56]	2019	Japan	147	Colorectal Cancer	rabbit anti-LAT1 antibody (1:5000 dilution)	ICH	scores of 4	OS,DFS	9
Sato, K. [51]	2019	Japan	245	ovarian carcinoma	rabbit anti-LAT1 antibody (1:800 dilution)	ICH	scores of 3, 4	OS,PFS	9
Sakata, T. [7]	2009	Japan	114	prostate cancer	mouse anti-LAT1 antibody (1:1 dilution)	ICH	scores of 4–9	OS	8

Abbreviations: ICH: Immunohistochemistry; NOS: Newcastle-Ottawa Scale; OS: overall survival; DFS: disease free survival; PFS: progression free survival.

### Statistical analysis

The correlation between LAT1 expression and prognosis was assessed by meta-analysis using studies determined eligible by the inclusion criteria. The outcome endpoints (OS, DFS and PFS) were calculated for pooled HRs and 95% CIs. Subgroup analysis was conducted when each subgroup has or exceeds three studies. Higgins’s I^2^ statistic and Cochran’s Q test were used to evaluate statistical heterogeneity [25], and lack of heterogeneity among studies was indicated when *P* value >0.10 and I^2^<40%. In accordance with heterogeneity status, we used the random effects model or fixed effects model to merge HR.

This analysis uses Funnel plots and the Egger’s test to assess any potential publication bias [26]. In the event of publication bias, the Duval and Tweedie’s trim and fill method was used to evaluate their impact on the overall effect [27]. Sensitivity analysis also identified potential outliers by omitting each study or specific studies. Stata 14.0 (StataCorp, College Station, TX) was used to conduct statistical analyses. The *P* values were two-tailed and statistical significant when *p*<0.05, except for those with statistical heterogeneity.

## Results

### Demographic characteristics

**Fig 1:**
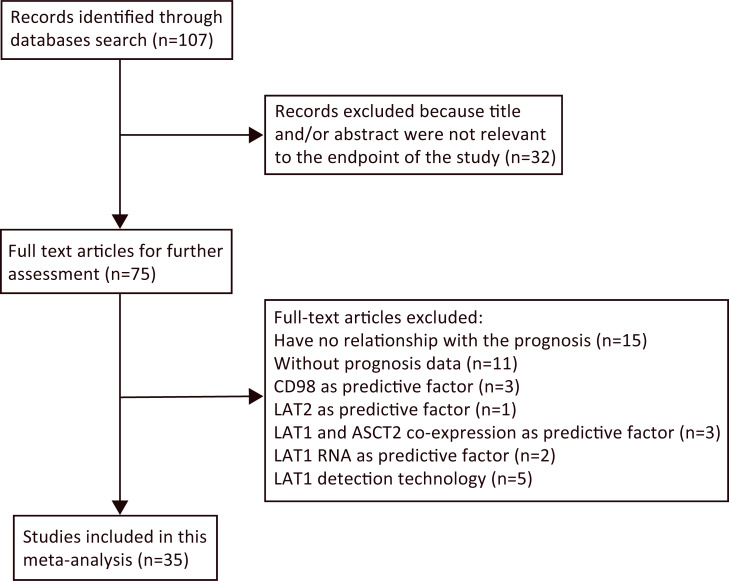
The flow chart of the selection process in our meta-analysis.

As shown in the search flowchart (Fig 1), we initially retrieved 107 records in document retrieval databases using a predefined search approach. After reviewing the retrieved titles and abstracts, we excluded 32 articles that described irrelevant endpoints of the 75 articles downloaded as full-text, we excluded 40 studies that were unrelated to prognosis (15 articles), had no prognosis data (11 articles), where the prognosis was related to CD98 (3 articles), LAT2 (1 article), LAT1 and ASCT2 co-expression (3 articles), status of LAT1 RNA in cancers(2 articles), or were a description about LAT1 detection technology(5 articles). The remaining 35 articles that reached our meta-analysis criteria were selected to evaluate LAT1 expression and solid cancer prognosis of patients. To assess the relevance between LAT1 and the prognosis of solid cancer, 4,579 eligible patients were selected for this analysis. Sample cases ranged from 21 to 321. Of the 35 prognostic studies, 35 were on OS, 5 on DFS, and 7 on PFS. Most studies were performed in Japan (n = 33), followed by China (n = 1) and Thailand (n = 1), respectively. As for the cancer type, 11 studies evaluated lung cancer, of which 10 were non-small cell lung cancer, pancreatic cancer and biliary tract cancer each have 3 studies, hepatocellular carcinoma, ovarian tumor, gastric cancer, prostatic cancer and colorectal cancer each have 2 studies, breast cancer, multiple myeloma, tongue cancer, laryngeal squamous cell carcinoma, cutaneous melanoma, cutaneous angiosarcoma, malignant pleural mesothelioma and adenoid cystic carcinoma each have 1 study.

### Evidence synthesis

**Fig 2:**
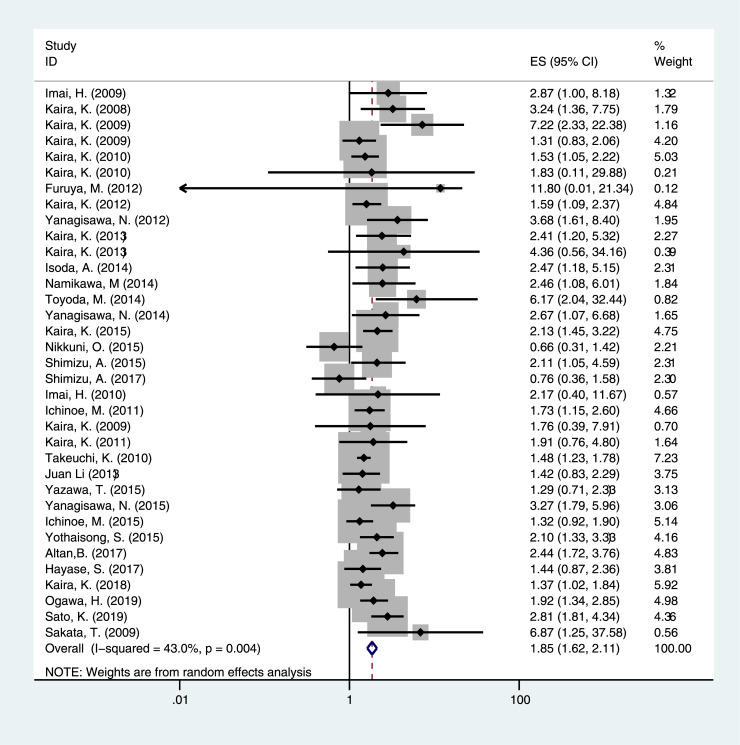
The correlation between LAT1 high expression and overall survival (OS) of solid cancers.

**Fig 3:**
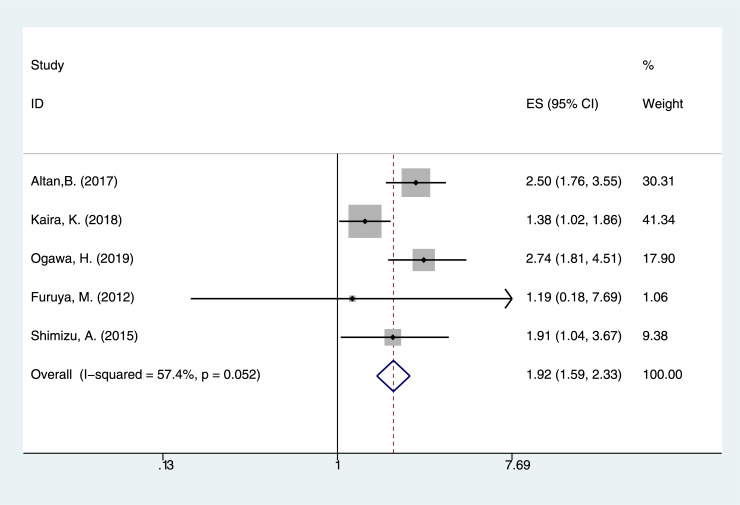
The correlation between LAT1 high expression and disease free survival (DFS).

**Fig 4:**
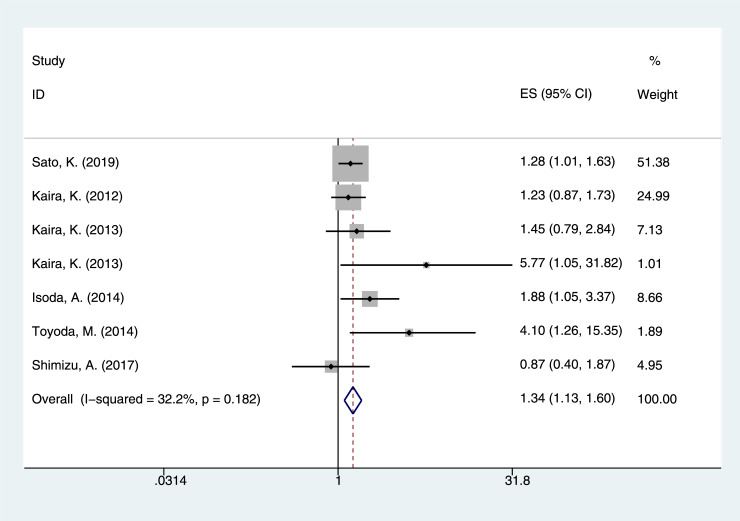
The correlation between LAT1 high expression and progression free survival (PFS).

This meta-analysis focused on the relationship between LAT1 status and the prognosis of cancer patients. In this study, the p value of heterogeneity test for OS was 0.004, and the I^2^ value was 43.0%. The random effect model was used. The results showed that there was a significant correlation between LAT1 high expression solid cancers and poor OS (pooled HR = 1.848, 95% CI = 1.620–2.108, *P <* 0.001) (Fig 2). Examining five DFS, the p value and I^2^ value of heterogeneity test report were 0.052 and 57.4%, respectively. The pooled HR and 95% confidence interval (CI) were calculated using the random effect model. The results suggested that LAT1 high expression was associated with poor DFS (pooled HR = 1.923, 95% CI = 1.585–2.333, *P <* 0.001) (Fig 3). Seven PFS databases were included in the meta-analysis, and the p value and I^2^ value of heterogeneity test report were 0.182 and 32.2%, respectively. The pooled HR and 95% confidence interval (CI) were calculated by using the fixed effect model. The results suggested that high LAT1 expression was associated with poor PFS (pooled HR = 1.345, 95% CI = 1.133–1.597, *P* = 0.001) (Fig 4).

To explore the source of heterogeneity, subgroup analysis was performed when more than 3 articles participated in each type. The results of subgroup analysis are shown in Table 2. In the cancer subgroups, there was a significant relationship between high LAT1 expression and poor OS in solid tumors observed in patients with lung cancer (HR = 1.573, 95% CI = 1.309–1.890, *P* < 0.001), non-small cell lung cancer (HR = 1.554, 95% CI = 1.345–1.794, P < 0.001), pancreatic cancer (HR = 2.052, 95% CI = 1.613–2.724, P < 0.001) and biliary tract cancer (HR = 2.253, 95% CI = 1.562–3.227, P < 0.001). When analyzed by the statistical method of LAT1 prognosis, the relationship was still found in the multivariate analysis (OS: HR = 2.052, 95% CI = 1.770–2.378, *P* <0.001; PFS: HR = 1.547, 95% CI = 1.089–2.199, *P* = 0.015; DFS: HR = 1.884, 95% CI = 1.196–2.968, *P* = 0.006) and univariate analysis (OS: HR = 1.439, 95% CI = 1.153–1.797, *P =* 0.001). Furthermore, subgroup analysis from cut off found that >10% of tumor cells (OS: HR = 1.786, 95% CI = 1.503–2.122, *P* <0.001), scores of 3, 4 (OS: HR = 1.955, 95% CI = 1.493–2.558, *P* <0.001; PFS: HR = 1.349, 95% CI = 1.027–1.773, *P* = 0.032), scores of 6–9 (OS: HR = 1.792, 95% CI = 1.141–2.812, *P* = 0.011), other cut off value (OS: HR = 2.277, 95% CI = 1.557–3.331, *P* <0.001), all of the results showed a poorer prognosis. Moreover, in the ethnicity subgroup, most of studies were from Asian, the results shown high LAT1 expression were all associated with poorer prognosis (OS: HR = 1.848, 95% CI = 1.620–2.108, *P* <0.001; PFS: HR = 1.345, 95% CI = 1.133–1.597, *P* = 0.001; DFS: HR = 1.923, 95% CI = 1.585–2.333, *P* <0.001).

**Table 2:** Hazard ratio for the association between LAT1 expression and solid tumor prognosis.

Analysis	No of patients	No of studies	HR(95%CI)	*P*	Heterogeneity	
					*I2* (%)	Ph
**All Studies**						
OS	4579	35	1.848(1.620–2.108)	**<0.001**	43.0%	0.004
PFS	540	7	1.345(1.133–1.597)	**0.001**	32.20%	0.182
DFS	748	5	1.923(1.585–2.333)	**<0.001**	57.40%	0.052
**cancer types**						
lung cancer	1966	11	1.573(1.309–1.890)	**<0.001**	23.60%	0.219
non-small cell lung cancer (OS)	1861	10	1.554(1.345–1.794)	**<0.001**	28.2%	0.185
pancreatic cancer(OS)	273	3	2.052(1.613–2.724)	**<0.001**	53.3%	0.117
biliary tract cancer(OS)	451	3	2.253(1.562–3.227)	**<0.001**	0.0%	0.882
**HR estimation**						
Multivariate analysis(OS)	3395	24	2.052(1.770–2.378)	**<0.001**	30.70%	0.078
Multivariate analysis(PFS)	421	4	1.547(1.089–2.199)	**0.015**	49.40%	0.231
Multivariate analysis(DFS)	380	3	1.884(1.196–2.968)	**0.006**	67.60%	0.046
Univariate analysis(OS)	1184	11	1.439(1.153–1.797)	**0.001**	40.80%	0.077
Univariate analysis(PFS)	436	3	1.323(0.734–2.383)	0.352	30.20%	0.138
**cut off**						
>10% of tumor cells(OS)	2386	16	1.786(1.503–2.122)	**<0.001**	17.80%	0.025
scores of 3, 4(≥26% of tumor cells)(OS)	1115	9	1.955(1.493–2.558)	**<0.001**	50.60%	0.039
scores of 3, 4(≥26% of tumor cells)(PFS)	648	7	1.349(1.027–1.773)	**0.032**	33.00%	0.188
scores of 6-9(OS)	217	3	1.792(1.141–2.812)	**0.011**	60.90%	0.078
other(OS)	683	5	2.277(1.557–3.331)	**<0.001**	37.60%	0.17
**Ethnicity**						
Asian(OS)	4579	35	1.848(1.620–2.108)	**<0.001**	43.0%	0.004
Asian (PFS)	540	7	1.345(1.133–1.597)	**0.001**	32.20%	0.182
Asian (DFS)	748	5	1.923(1.585–2.333)	**<0.001**	57.40%	0.052

Abbreviations: OS: overall survival; DFS: disease free survival; PFS: progression free survival; HR: hazard ratio; CI: confidence interval.

### Publication bias and sensitivity analysis

**Fig 5:**
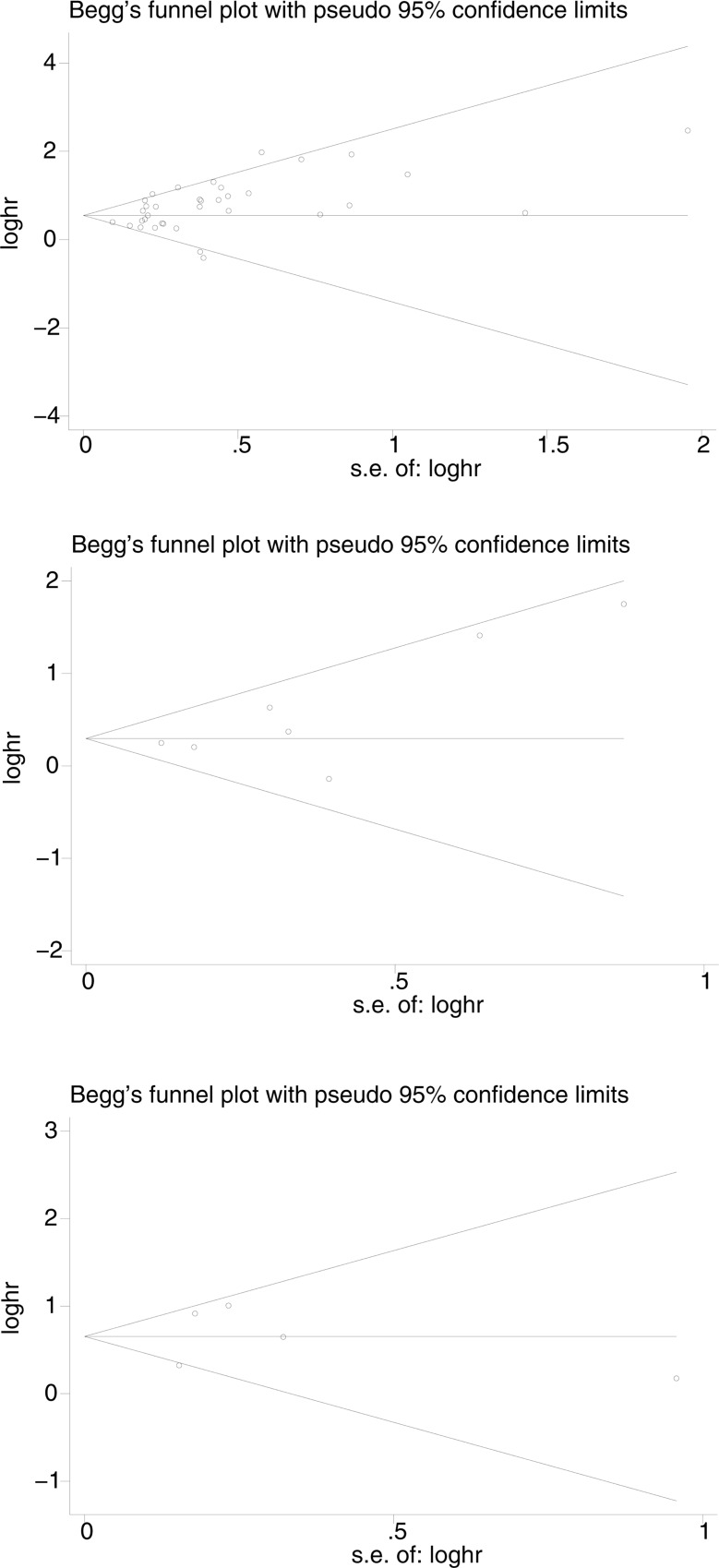
Begg’s funnel plots for the studies involved in the meta-analysis. A. overall survival B: progression free survival. C. disease free survival. Abbreviations: loghr, logarithm of HRs; s.e., standard error.

**Fig 6:**
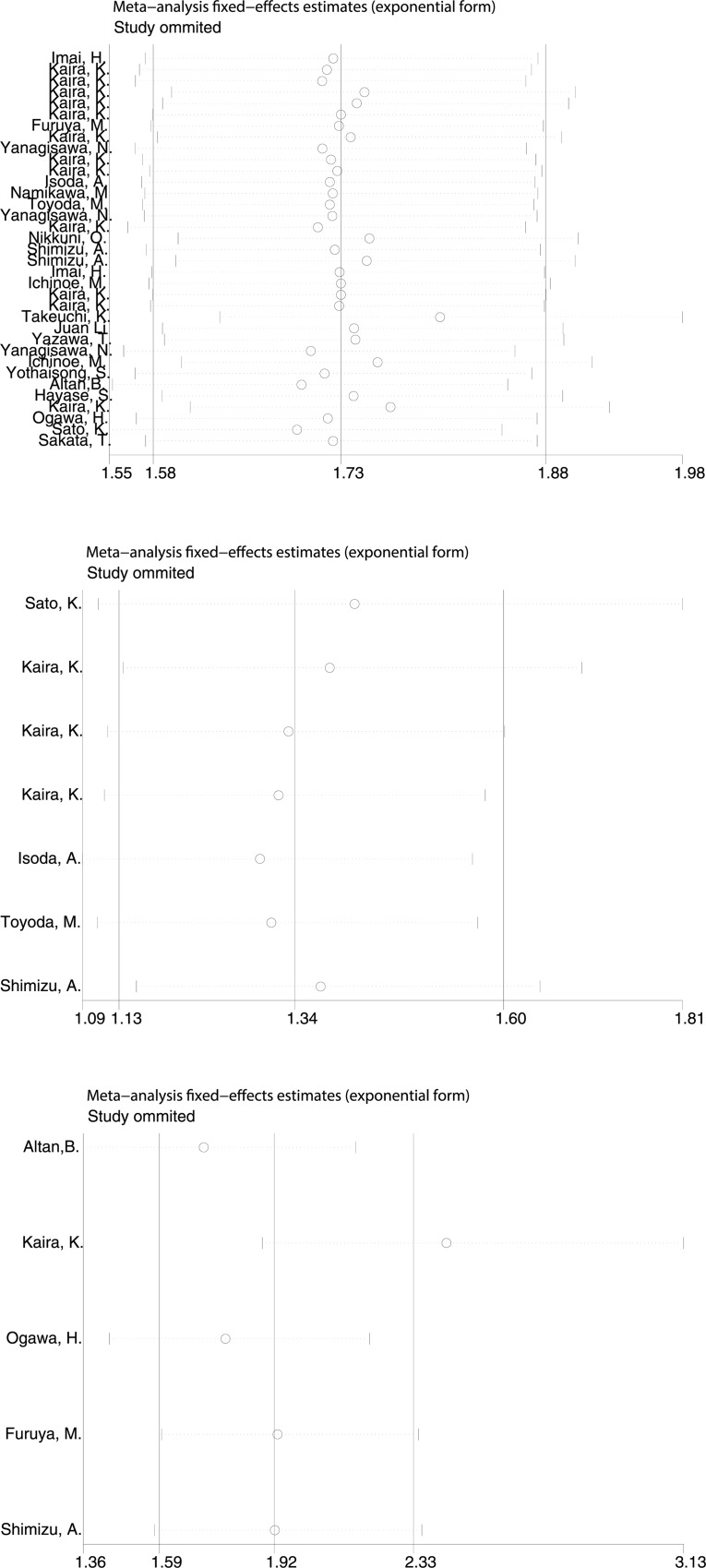
Sensitivity analysis of the meta-analysis. A. overall survival B: progression free survival. C disease free survival.

Begg’s funnel plot and Egger’s test were used to detect publication bias of the above literature. There was no evidence of significant asymmetry on the funnel plot, and Egger's test found no publication bias (p<0.05) (Fig 5). Our sensitivity analysis confirmed the impact of these results. None of the individual studies had guided this meta-analysis and eliminating any single study had no significant effect on the final results (Fig 6).

## Discussion

There is increasing evidence showing that LAT1 plays a significant role in promoting tumor cell growth and proliferation [2, 3, 28, 29]. By interfering with the expression of LAT1, Hayashi K et al. demonstrated that the proto-oncogene c-Myc binds to the LAT1 promoter sequence to accelerate its expression [30]. Furthermore, the heterodimeric complex formed by the binding of LAT1 and CD98 plays a significant role in integrin signaling of cancer cells, sequentially activating the phosphatidylinositide 3-kinase/AKT (PI3K/AKT), p130CAS and focal adhesion kinase (FAK) signaling pathway [31, 32]. The relationship of LAT1 expression levels and prognosis of solid cancer patients is difficult to determine from individual clinical reports, as they involve small sample sizes.

To our knowledge, this is the first complete study demonstrating the prognosis significance of LAT1 status in solid cancers. In this meta-analysis, we examined 16 cancer types, including lung cancer [16, 20, 33–41], pancreatic cancer [42–44], biliary tract cancer [45–47], hepatocellular carcinoma [48, 49], ovarian tumor [50, 51], breast cancer [8], gastric carcinomas [52, 53], prostatic cancer [7, 54], colorectal cancer [55, 56], adenoid cystic carcinoma [17], multiple myeloma [57], tongue cancer [58], laryngeal squamous cell carcinoma [59], cutaneous melanoma [60], cutaneous angiosarcoma [61] and malignant pleural mesothelioma [62]. We assessed the survival data of 4,579 cancer patients with regard to LAT1 status, and 35 different studies were systematically included. In summary, the results clearly indicate that LAT1 overexpression is an indicator for poor prognosis in solid cancer, with poor OS (pooled HR = 1.848, 95% CI = 1.620–2.108, P < 0.001), poor DFS (pooled HR = 1.923, 95% CI = 1.585–2.333, P < 0.001) and poor PFS (pooled HR = 1.345, 95% CI = 1.133–1.597, P = 0.001).

In our paper, we also used subgroup analysis to illustrate the relationship between high expression of LAT1 and prognosis. The subgroup analysis was conducted by at least 3 articles participated in each type including cancer type, HR estimation, cut off value and ethnicity. The significant relationship was still found in these subgroup.

The results of our current analysis may be affected by a number of limitations. First, the meta-analysis may be influenced by the exclusion of articles that are not published in English as well as articles that are proceedings or books. Despite our rigorous search and attempts to obtain unpublished information, some data loss was inevitable. Second, most of the studies involved were retrospective, with positive results more likely to be published than negative. Third, although the expression of LAT1 was detected by immunohistochemistry in all studies, the antibody concentration and cut-off value were different for each study, which could lead to some deviation in the combined analysis. Lastly, LAT1 is typically co-expressed with CD98, which may play a synergistic role in the development and progression of tumors.

Despite these limitations, this meta-analysis has several advantages. This is the first comprehensive statistical study of LAT1 that evaluates the prognostic significance of LAT1 status in solid cancers before treatment. We collected 4,579 patients from the limited subsets, which represented a large number of cases and significantly improved the statistical value in this analysis. Additionally, no publication biases were detected, suggesting that the results of the analysis are impartial.

The results suggest that before tumor treatment, the expression of LAT1 can be used to predict tumor patient outcome. Nevertheless, to verify our finding more accurately, more comprehensive studies will be required to evaluate the predictive value of high LAT1 expression for future clinical practice.

## Supporting information

S1 Checklist PRISMA 2009 checklist. (DOC)
